# Extra-corporeal-cardiopulmonary-resuscitation vs. conventional-cardiopulmonary-resuscitation: an in-depth look into short- and long-term neurological outcomes

**DOI:** 10.1186/s13019-025-03708-z

**Published:** 2025-12-24

**Authors:** Farah Yasmin, Asad Ur Rab, Afia Salman, Muhammad Ahmed Ali Fahim, Hafsah Alim Ur Rahman, Abdul Moeed, Eman Ali, Muhammad Sohaib Asghar, Iqbal Ratnani, Salim Surani

**Affiliations:** 1 Yale School of Medicine, New Haven, CT USA; 2 Foundation University Medical College, Islamabad, Pakistan; 3 Dow University of Health Sciences, Karachi, Pakistan; 4 Advent Health, Sebring, FL USA; 5 Houston Methodist Hospital, Houston, TX USA; 6 Texas A&M University, TX, USA

**Keywords:** Cardiopulmonary resuscitation, Extracorporeal cardiopulmonary resuscitation, Cerebral performance categories

## Abstract

**Background:**

Extracorporeal cardiopulmonary resuscitation (eCPR) is a new approach for cardiac arrest management and is regarded as a possible alternative for conventional cardiopulmonary resuscitation (cCPR).

**Aim:**

Our systematic review and meta-analysis seek to compare outcomes of cCPR versus eCPR in patients with out-of-hospital cardiac arrest (OHCA).

**Methods:**

Preferred Reporting Items for Systematic Reviews and Meta-Analyses (PRISMA) guidelines were followed in this study and the questionnaire utilized the PICO (Patient, Intervention, Control, Outcome) framework. A search strategy using MeSH (Medical Subject Headings) terms was made from which Pubmed/Medline, SCOPUS, and Cochrane Library were searched from inception till March 2024 to reveal all relevant studies. Conference proceedings, www.clinicaltrials.gov, and bibliometrics of published articles were also searched for gray literature.

**Results:**

Compared with conventional CPR (cCPR), extracorporeal CPR (eCPR) was associated with higher odds of favorable neurological outcome at discharge (OR 2.61, 95% CI 1.28–5.32; *p* = 0.008; I²=82%), 3-months (OR 3.29, 95% CI 1.63–6.63; *p* = 0.0009; I²=46%), and 6-months (OR 1.97, 95% CI 1.24–3.12; *p* = 0.004; I²=12%). The increase at 1-month was not significant (OR 2.15, 95% CI 0.87–5.34; *p* = 0.10; I²=90%). eCPR also improved survival-to-discharge (OR 1.84, 95% CI 1.17–2.92; *p* = 0.009; I²=73%).

**Conclusion:**

eCPR in the management of OHCA patients has more favorable neurological and survival outcomes as compared to cCPR.

**Supplementary Information:**

The online version contains supplementary material available at 10.1186/s13019-025-03708-z.

## Introduction

According to the American Heart Association (AHA) and the American College of Cardiology, cardiac arrest is the abrupt stoppage of the heart’s pumping function, resulting in unconsciousness, absence of breathing, and no detectable pulse. Without prompt intervention, cardiac arrest can lead to sudden death [1]. The majority of cardiac arrests result from a malfunction in the electrical system of a diseased heart, leading to abnormal rhythms like ventricular tachycardia or ventricular fibrillation. Additional causes include heart tissue scarring, thickening of the heart muscle (cardiomyopathy), certain medications, electrical and blood vessel abnormalities, recreational drug use, and commotio cordis [2]. Insufficient blood flow to the brain and other organs during cardiac arrest can result in unconsciousness, disability, or death without prompt intervention [3]. Every year, over 400,000 Americans die from sudden cardiac death [1]. 

Cardiac arrests are commonly classified into two groups based on where they occur: in-hospital cardiac arrest (IHCA) and out-of-hospital cardiac arrest (OHCA). Research indicates that OHCA patients generally experience poorer outcomes than IHCA patients [4]. OHCA is a major cause of death in developed countries. The average global incidence of OHCA in adults is 95.9 per 100,000 people per year [5]. 

Historically, conventional cardiopulmonary resuscitation (cCPR) involving chest compressions—pushing down at least two inches in the center of the chest at a rate of 100 to 120 pushes per minute, allowing the chest to fully recoil after each compression—has been regarded as the optimal approach for managing cardiac arrest patients [6]. A novel approach to treating cardiac arrest is extracorporeal cardiopulmonary resuscitation (eCPR), which involves using veno-arterial extracorporeal membrane oxygenation (VA-ECMO) in patients who experience sudden, unexpected loss of pulse due to cardiac mechanical activity cessation. eCPR is now acknowledged in guidelines by the Extracorporeal Life Support Organization (ELSO) and AHA as a viable option for selected patients in cardiac arrest [7]. 

Several studies have compared cCPR to extracorporeal methods, but the effectiveness of eCPR compared to conventional treatment for patients with refractory OHCA is still uncertain [8]. Our systematic review and meta-analysis seeks to compare outcomes of cCPR versus eCPR in patients with OHCA. This article will assist clinicians by presenting current, evidence-based information on the efficacy of eCPR compared to cCPR. By consolidating existing research, this article can guide healthcare providers in making more informed decisions about the optimal approach, potentially resulting in enhanced outcomes for patients with cardiac arrest.

## Methods

This systematic review and meta-analysis was performed in accordance with the Preferred Reporting Items for Systematic Reviews and Meta-analysis (PRISMA) [9] and Cochrane Collaboration guidelines with a PRISMA checklist being provided in Supplementary Table 1 [10]. The review protocol was prospectively registered with the Research Registry (Registry of Systematic Reviews/Meta-Analyses; ID: reviewregistry2032).

### Literature search and study selection

Databases such as MEDLINE, SCOPUS, and the Cochrane Library were searched from inception till August 2025. The following keywords were used in the search string: “pre-hospital”, “out-of-hospital”, “cardiopulmonary resuscitation”, “cardiopulmonary arrest”, “cardiac arrest”, “extracorporeal cardiopulmonary resuscitation”, “extracorporeal support”, and “extracorporeal membrane oxygenation”. The detailed search string is shown in Supplementary Table 2. Additionally, conference proceedings, www.clinicaltrials.gov, and bibliometrics of published articles were browsed to ensure no articles from the gray literature were missed.

Articles from the literature search were exported to Endnote Reference Library (Version X7.5; Clarivate Analytics, Philadelphia, Pennsylvania) software, where the duplicates were identified and removed. The remaining articles were then thoroughly reviewed by independent reviewers (E.A and A.M), ensuring that the selected articles met the defined eligibility criteria. The following inclusion criteria were used to shortlist studies: [1] studies reporting survival or neurological outcomes [2], adult patients (≥ 18 years) [3], experiencing out-of-hospital cardiopulmonary arrest (OHCA) [4], comparison between cCPR and eCPR. Studies were excluded that: [1] did not mention OHCA outcomes separately and reported combined in-and out-hospital outcomes [2], included pediatric patients [3], focused on hypothermic cardiac arrests [4], studies published in language other than English, and [5] studies with small sample size (*n* < 10).

### Outcomes

The primary outcome of our study was to determine the occurrence of favorable neurological outcomes at the time of discharge, as well as at 1, 3, and 6 months after the cardiac arrest. The neurological condition was assessed using the Cerebral Performance Categories (CPC) [11], a widely used five-point scale for evaluating neurological states following a cardiac arrest. CPC 1 represented good cerebral performance, involving normal neurological function or mild neurological or psychological dysfunction, while CPC 2 denoted sufficient cerebral function for independent daily activities. These two categories, CPC 1, and CPC 2 were considered favorable neurological outcomes [12]. Our secondary outcomes, on the other hand, were the examination of survival until discharge from the hospital.

### Data extraction and quality assessment

Data extraction was extracted and verified by two reviewers (E.A. and A.M.). Any discrepancies were resolved through discussion and consensus. Further random ball sampling was done to include all the relevant studies. Data extracted from each study included: study design, study population, sample size, number of patients in each group, general patient characteristics (age and gender), and primary and secondary endpoints. The quality assessment of randomized controlled trials (RCTs) was done using the risk of bias-2 tool (RoB-2) of Cochrane Collaboration [13]. The Newcastle-Ottawa Scale (NOS) was utilized for the risk of bias evaluation of non-randomized studies [14] with grading scores ≥ 7 deemed to be low risk of bias. We assessed certainty using GRADE across five domains: risk of bias, inconsistency, indirectness, imprecision, and publication bias. Randomized evidence started as high and observational as low, with downgrades/upgrades applied; two reviewers graded each outcome in GRADEpro, resolved disagreements by consensus, and reported final ratings as high, moderate, low, or very low. For outcomes with ≥ 10 studies, publication bias was assessed through visual inspection of funnel plots.

### Statistical analysis

Review Manager (version 5.4.1; Copenhagen: The Nordic Cochrane Centre, The Cochrane Collaboration, 2020) was used for all relevant meta-analyses of this study. A random-effects model was utilized for both and for meta-analysis odds ratios (ORs) were derived and collected along with their 95% Confidence Intervals (CI) from dichotomous data. A *P*-value < 0.05 was considered statistically significant for all outcomes. Heterogeneity was assessed with Higgin’s I^2^ test. A value of I^2^ = 25%−50% was considered mild, 50%−75% as moderate, and > 75% as significant heterogeneity.

For outcomes with substantial heterogeneity (I² >75%), we performed a leave-one-out sensitivity analysis, sequentially omitting each study to assess its impact on the pooled effect and heterogeneity (I²).

## Results

**Fig. 1:**
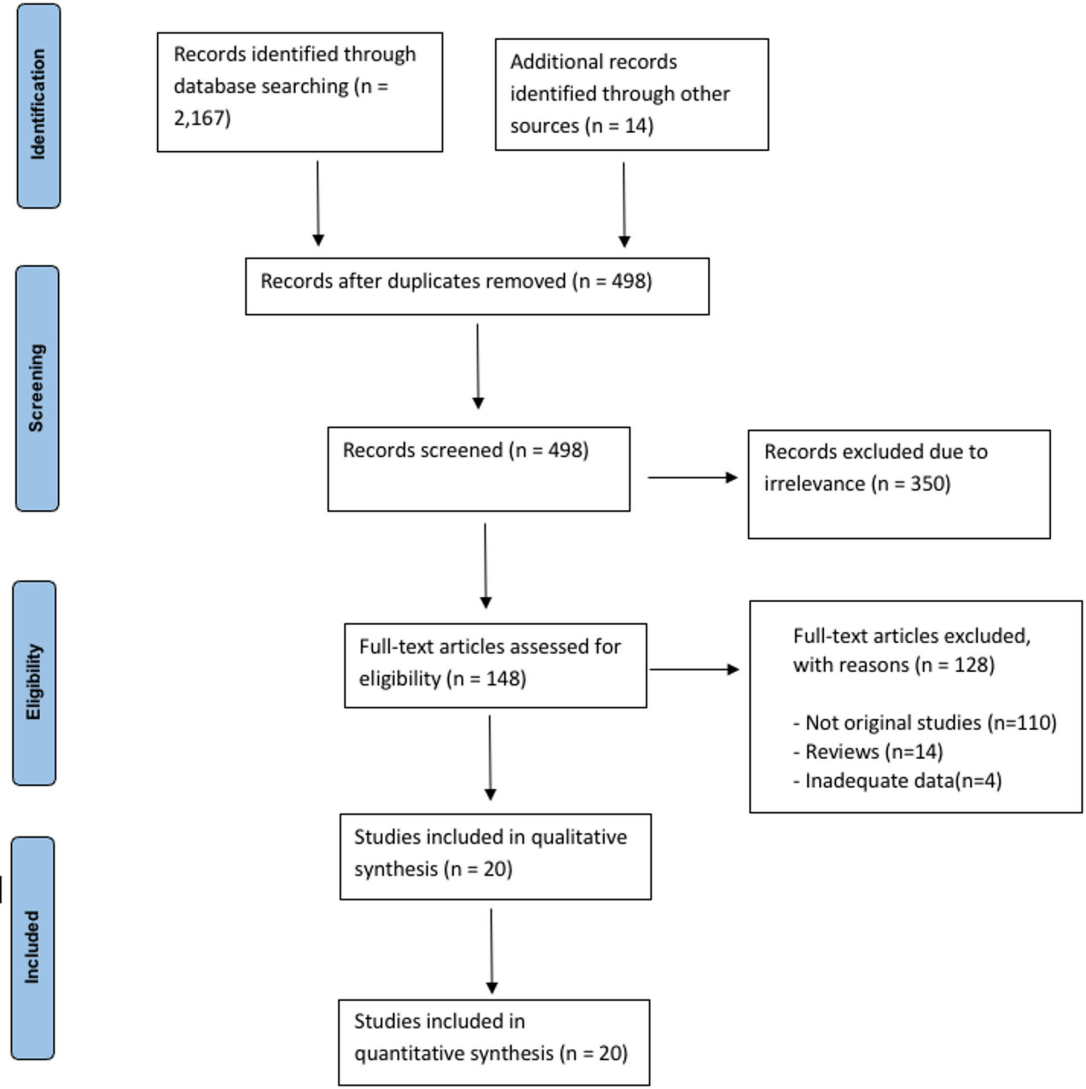
PRISMA Flowchart

Following a detailed search of the aforementioned databases, 20 studies [15–34]were identified and included in the meta-analysis. A detailed overview of the selection process is presented in the PRISMA flow diagram (Fig. 1). The included studies comprised a total of 20,178 patients, of whom 2,595 were allocated to the eCPR group and 17,583 to the cCPR group. Study characteristics and baseline patient characteristics are summarized in Tables 1 and 2, respectively.

**Table 1:** Study characteristics and outcome measures of eligible studies

Study	Year of Publication	Location	Study Design	Sample Size *n* (eCPR/cCPR)	Primary Outcome(s)	Secondary Outcome(s)
Bougoin et al.	2020	France	Retrospective Study	525/12,666	Survival to hospital discharge	Neurological outcomes at hospital discharge
Choi et al.	2016	Korea	Retrospective Study	10/50	Neurological outcomes at 1 month	Survival at 1 month
Choi et al.	2016	Korea	Retrospective Study	320/320	Neurological outcomes at hospital discharge	Survival to hospital discharge
Jeong et al.	2022	Korea	Retrospective Study	271/271	Favorable neurological survival	-
Kim et al.	2014	Korea	Retrospective Study	52/52	Neurological outcomes at 3 months	Survival at 24 h, hospital discharge, and 3 months
Kitada et al.	2020	Japan	Retrospective Study	307/2278	Neurological outcomes at 1 month	-
Maekawa et al.	2013	Japan	Retrospective Study	24/24	Neurological outcomes at 3 months	-
Nakashima et al.	2019	Japan	Retrospective Study	250/157	Neurological outcomes at 6 months	Survival at 6 months
Patricio et al.	2019	Belgium	Retrospective Study	49/50	Neurological outcomes at 3 months Survival to ICU discharge	-
Sakamoto et al.	2014	Japan	Prospective Study	260/194	Neurological outcomes at 1 and 6 months	-
Schober et al.	2017	Austria	Retrospective Study	7/232	Neurological outcomes at 6 months	-
Sun Peng et al.	2023	China	Retrospective Study	33/100	Neurological outcomes at 3 months	-
Suveiren et al.	2023	The Netherlands	Randomized Controlled Trial	70/64	Favorable neurological survival at 1 month	Neurological outcomes at 6 months Survival outcomes at 1 and 6 months
Verdonschot et al.	2024	The Netherlands	Retrospective Study	23/60	The proportion of patients requiring cCPR who would be treated with eCPR	Hospital survival outcomes Glasgow Coma Scale score Regain of consciousness
Yannopoulos et al.	2017	USA	Prospective Study	62/170	Neurological outcomes at discharge	Neurological outcomes at 3 months
Yannopoulos et al.	2020	USA	Randomized Controlled Trial	15/15	Survival to Hospital Discharge	Survival outcomes at 1, 3, and 6 months Neurological outcomes at hospital discharge, 1, 3, and 6 months
Yoshida et al.	2020	Japan	Retrospective Study	38/493	Neurological outcomes at 1 and 3 months	Survival outcomes at 1 and 3 months
Shih et al.	2024		Retrospective Study	77/154	Neurological outcomes at discharge	Survival outcomes at 1 month and hospital discharge
Lee et al.	2025	Korea	Retrospective Study	78/101	Neurological outcomes at discharge	Survival outcomes at 1 month
Belohlavek et al.	2022	Czech Republic	Randomized Controlled Trial	124/132	Neurological outcomes at 6 months	Neuorlogical outcomes at 1 month Survival outcomes at 1 month

n: number of participants eCPR: extracorporeal cardiopulmonary resuscitation; cCPR: conventional cardiopulmonary resuscitation; ICU: Intensive Care Unit

**Table 2:** Baseline characteristics

	Author, year	Bougoin 2020	Choi 2016	Choi 2016	Kim 2014	Kitada 2020	Maekawa 2013	Nakashima 2019	Patricio 2019	Sakamoto 2014	Schober 2017	Yannopoulos 2017	Yannopoulos 2020	Yoshida 2020	Jeong 2022	Suveiren 2023	Sun Peng 2023	Verdonschot 2024	Shih 2024	Lee 2025	Belohlavek 2022
**Sample size n**	**eCPR**	525	10	320	52	307	24	250	49	260	7	62	15	38	271	70	33	23	77	78	124
	**cCPR**	12,666	50	320	52	2278	24	157	50	194	232	170	15	493	271	64	100	60	154	101	132
**Age**,** years**	**eCPR**	50 (13)	58 (6)	56 (7)	53 (8)	60 (6)	56 (4)	58 (5)	-	56 (NR)	46 (8)	58 (10)	59 (10)	61 (16)	58 (49–67)	54 ± 12	57.1 ± 11.9	46.0 (33.5–51.5)	57.0 (47.0–65.0)	54 (39.0–65.0)	59 (48–66)
	**cCPR**	66 (16)	59 (12)	58 (6)	55 (8)	76 (5)	58 (5)	60 (5)	-	58 (NR)	60 (6)	56 (7)	58 (11)	72 (16)	57 (47–71)	57 ± 10	64.1 ± 12.4	59.5 (51.8–65.0)	56.0 (44.0–66.0)	54 (39.0–65.0)	57 (47–65)
**Male n(%)**	**eCPR**	441 (84)	7 (70)	258 (81)	40 (77)	257 (84)	19 (79)	227 (91)	-	235 (90)	5 (71)	44 (71)	14 (93)	27 (71)	211 (77.9)	63 (90)	26 (78)	19 (82.6%)	63 (81.8)	66 (55.0–72.0)	102 (82)
	**cCPR**	8486 (67)	38 (76)	259 (81)	38 (73)	1457 (64)	19 (79)	139 (89)	-	172 (89)	173 (75)	124 (73)	11 (73)	307 (62)	206 (76.0)	57 (89)	85 (85)	46 (76.7%)	124 (80.5)	89 (88.1)	110 (83)
**DM n(%)**	**eCPR**	-	-	-	11 (21)	-	-	-	-	-	0 (0)	12 (19)	3 (20)	-	66 (27.7)	10/62 (16)	12 (36.4)	-	21 (27.3)	18 (23.1)	19/104 (18)
	**cCPR**	-	-	-	6 (12)	-	-	-	-	-	44 (19)	37 (22)	3 (20)	-	58 (25.0)	6/54 (11)	26 (26)	-	27 (17.5)	17 (16.8)	17/83 (21)
**HTN n(%)**	**eCPR**	-	-	-	13 (25)	-	-	-	-	-	2 (28)	30 (48)	2 (13)	-	94 (39.0)	24/44 (55)	18 (54)	-	24 (31.2)	24 (30.8)	47/104 (44)
	**cCPR**	-	-	-	12 (23)	-	-	-	-	-	67 (29)	63 (37)	5 (33)	-	88 (37.6)	15/33 (45)	45 (45)	-	33 (21.4)	42 (41.6)	42/83 (51)
**CAD n(%)**	**eCPR**	-	-	-	15 (29)	-	-	-	-	-	1 (14)	6 (9)	2 (13)	-	-	7/61 (12)	12 (36.4)	9 (39.1)	13 (16.9)	11 (14.1)	17/104 (16)
	**cCPR**	-	-	-	11 (21)	-	-	-	-	-	65 (28)	24 (14)	4 (27)	-	-	6/53 (11)	34 (34)	33 (55.0)	18 (11.7)	19 (18.8)	17/83 (21)
**ACS n(%)**	**eCPR**	194 (37)	-	-	36 (69)	-	-	163 (65)	-	165 (64)	-	-	-	8 (21)	-	10/61 (16)	-	-	-	-	-
	**cCPR**	196 (37)	-	-	9 (17)	-	-	82 (52)	-	114 (59)	-	-	-	20 (4)	-	10/55 (18)	-	-	-	-	-
**PE n(%)**	**eCPR**	16 (3)	-	-	2 (4)	-	-	-	-	-	-	-	-	10 (26)	-	1 (1)	-	5 (21.7)	-	-	-
	**cCPR**	18 (3)	-	-	1 (2)	-	-	-	-	-	-	-	-	10 (2)	-	1 (1)	-	4 (6.7)	-	-	-
**VT/VF n(%)**	**eCPR**	357 (68)	3 (30)	93 (29)	31 (60)	215 (70)	13 (54)	250 (100)	-	260 (100)	4 (57)	62 (100)	15 (100)	0 (0)	-	-	-	-	-	65 (83.3)	72 (58)
	**cCPR**	3167 (25)	13 (26)	90 (28)	29 (56)	-	14 (58)	157 (100)	-	194 (100)	135 (58)	170 (100)	15 (100	0 (0)	-	-	-	-	-	24 (23.8)	84 (64)
**Bystander CPR n(%)**	**eCPR**	425 (81)	8 (80)	96 (30)	22 (42)	157 (51)	13 (54)	115 (46)	-	127 (49)	2 (28)	52 (84)	13 (87)	-	148 (54.6)	-	-	18 (78.3%)	44 (57.1)	49 (62.8)	123 (99)
	**cCPR**	6206 (49)	41 (82)	74 (32)	16 (31)	1002 (44)	14 (58)	68 (43)	-	90 (46)	72 (31)	128 (75)	12 (80)	-	144 (53.1)	-	-	51 (85.0%)	73 (47.4)	48 (47.5)	129 (98)
**Time to Hospital (min)**	**eCPR**	-	14 (10)	19 (-)	-	-	31 (3)	34 (4)	-	30 (-)	42 (11)	-	48 (-)	11 (5)	-	-	-	-	23.0 (20.0–26.0)	18 (12.0–30.0)	-
	**cCPR**	-	19 (8)	19 (-)	-	-	28 (3)	32 (4)	-	31 (-)	56 (9)	-	50 (-)	16 (4)	-	-	-	-	22.0 (19.0–26.0)	20 (11.0–24.0)	-
**Low-Flow Time (min)**	**eCPR**	-	-	54(-)	70 (-)	-	51 (-)	55 (5)	-	-	93 (-)	-	-	39 (6)	-	-	-	63.0 (46.0–80.0)	-	47 (34.0–68.0)	-
	**cCPR**	-	-	47 (-)	68 (-)	-	52 (-)	-	-	-	78 (-)	-	59 (-)	-	-	-	-	55.0 (35.5–61.0)	-	43 (30.0–57.0)	-
**TTM n(%)**	**eCPR**	-	6 (60)	95 (30)	14 (27)	-	9 (38)	-	-	162 (63)	3 (43)	-	15 (100)	-	87 (32.1)	-	-	-	51 (66.2)	-	21 (17)
	**cCPR**	-	10 (67)	34 (11)	12 (60)	-	-	22 (46)	-	-	48 (55)	-	2 (100)	-	93 (34.3)	-	-	-	14 (9.1)	-	12 (9)
**CABG**,** PCI n(%)**	**eCPR**	159 (54)	5 (56)	-	29 (56)	-	5 (21)	-	-	97 (37)	2 (28)	46 (74)	-	-	-	34 (49)	-	-	-	65 (83.3)	72 (58)
	**cCPR**	966 (20)	2 (13)	-	3 (15)	-	-	16 (37)	-	-	11 (12)	-	2 (100)	-	-	14 (22)	-	-	-	24 (23.8)	84 (64)

n: number of participants; cCPR: conventional cardiopulmonary resuscitation; eCPR: extracorporeal cardiopulmonary resuscitation DM: diabetes mellitus; HTN: hypertension; CAD: coronary artery disease; ACS: acute coronary syndrome; PE: pulmonary embolism; VT/VF: ventricular tachycardia/ventricular fibrillation; min: minutes; TTM: targeted temperature management; CABG: coronary artery bypass grafting; PCI: percutaneous coronary intervention;

### Quality assessment, GRADE assessment and publication bias

All observational studies demonstrated low risk of bias on the Newcastle–Ottawa Scale, whereas randomized trials, assessed with RoB 2, showed an overall judgment of “some concerns.” (Supplementary Tables 3 and Supplementary Fig. 1) A GRADE assessment of this review indicated that all neurological outcomes were rated as ‘high’ certainty of evidence, whereas-survival-to-hospital discharge was rated as ‘moderate.’ Detailed results are provided in Supplementary Table 4. Publication bias was assessed graphically using funnel plots of log(OR) versus standard error for the outcome of survival-to-discharge (Supplementary Fig. 2). On visual inspection, the funnel plot appeared asymmetric, with a relative paucity of smaller studies on one side of the pooled effect, suggesting potential small-study effects/publication bias; given the limited number of studies, formal tests were underpowered and could not be performed.

### Primary outcomes

**Fig. 2:**
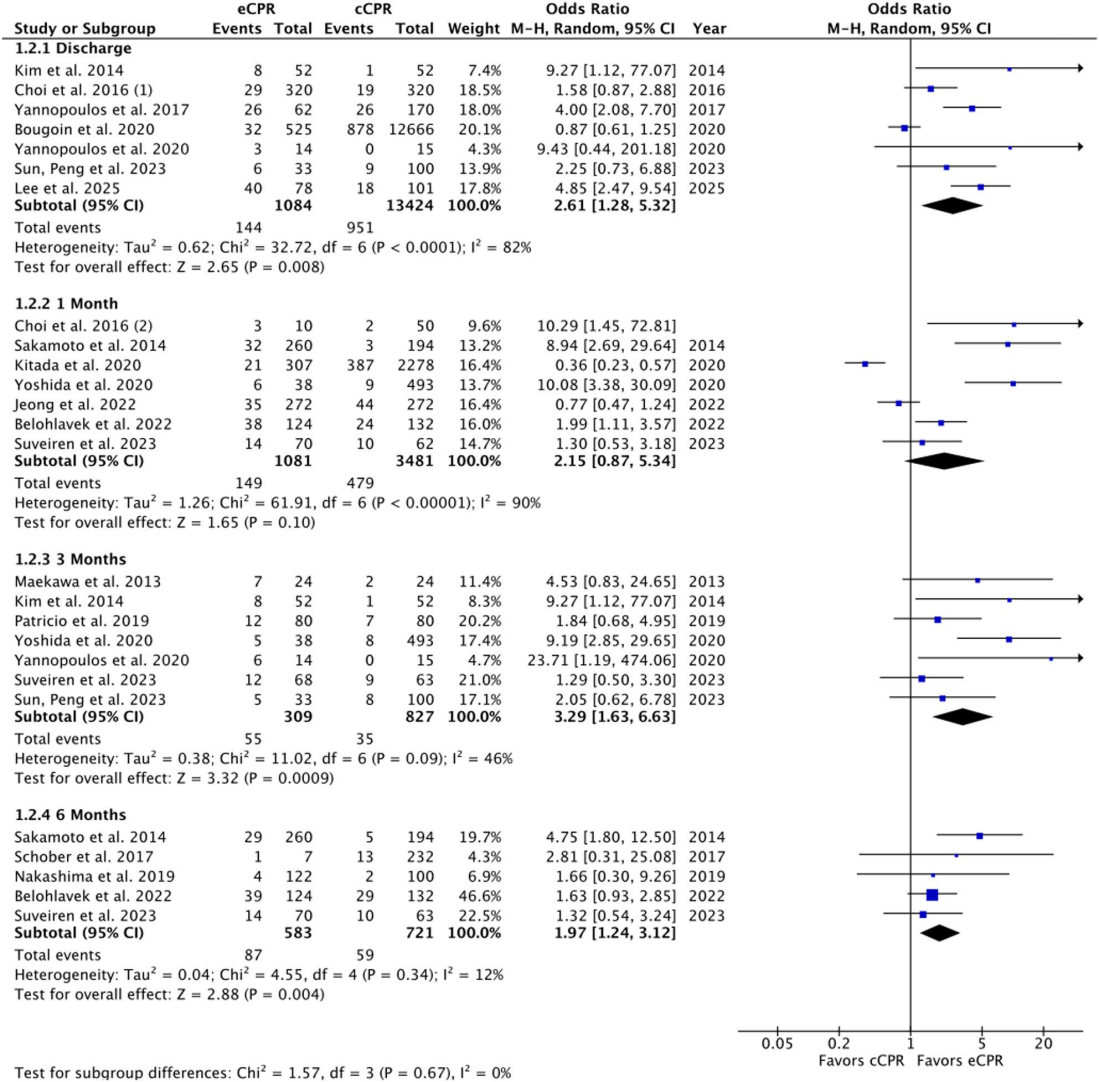
Forest Plots for Favorable Neurological Outcome at discharge, 1-month, 3-months, and 6-months

Across seven studies, extracorporeal CPR (eCPR) was associated with higher odds of favorable neurological outcome at discharge (OR 2.61, 95% CI 1.28–5.32; *p* = 0.008; I²=82%, substantial heterogeneity), 3-months (OR 3.29, 95% CI 1.63–6.63; *p* = 0.0009; I²=46%, moderate), and 6-months (OR 1.97, 95% CI 1.24–3.12; *p* = 0.004; I²=12%, low). At 1-month, the effect was directionally favorable but not significant (OR 2.15, 95% CI 0.87–5.34; *p* = 0.10; I²=90%, substantial). Forest plots for primary outcomes are shown in Fig. 2.

### Secondary outcomes

**Fig. 3:**
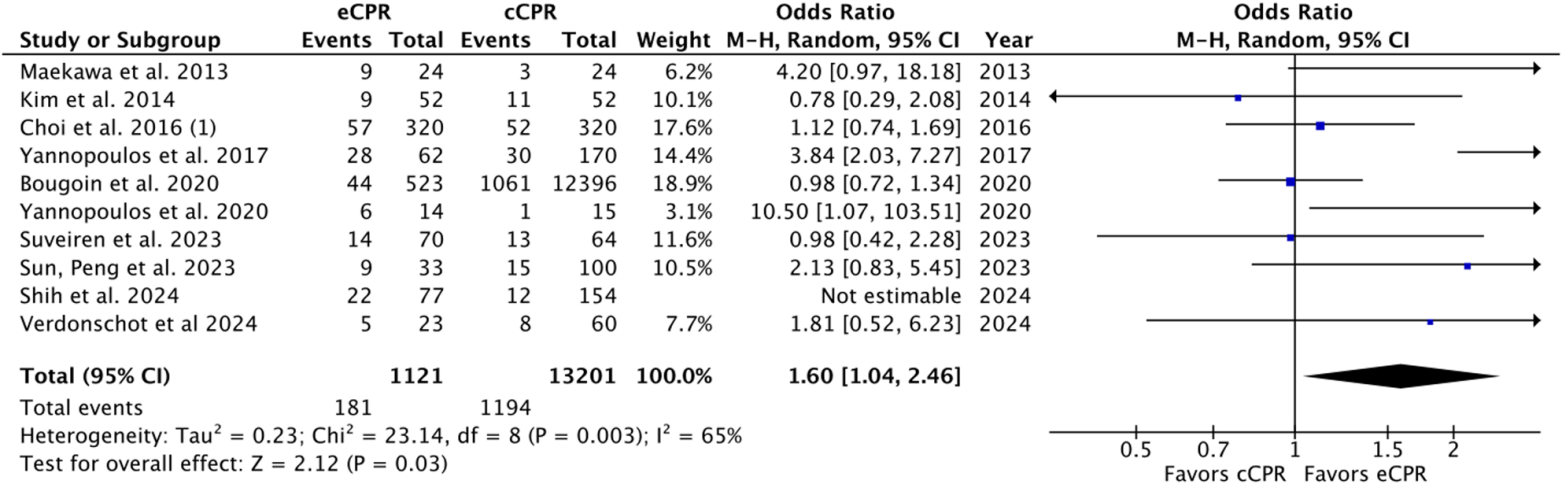
Forest Plot for Survival till discharge

Ten studies reported survival to discharge; eCPR was associated with higher odds versus cCPR (OR 1.84, 95% CI 1.17–2.92; *p* = 0.009; I²=73%, moderate–high heterogeneity). Forest plots for secondary outcomes appear in Fig. 3.

### Sensitivity analysis

For favorable neurological outcomes, heterogeneity decreased at discharge (I² from 77% to 44%) and at 1-month (I² from 91% to 84%) following leave-one-out analyses. Detailed results are provided in Supplementary Fig. 3 and Supplementary Tables 5–6.

## Discussion

Our systematic review and meta-analysis of 20 studies comparing the neurological outcomes of cCPR and eCPR reported that eCPR was associated with more favorable neurological outcomes and higher survival until discharge in patients experiencing OHCA. This comparison was statistically significant for all the outcomes except for favorable neurological outcomes at 1 month. However, the significant heterogeneity of studies in the assessment of favorable neurological outcomes at discharge and favorable neurological outcomes at 1 month may not provide a reliable estimate of the true effect size.

The utilization of rapid deployment veno-arterial oxygenation in eCPR promotes systemic circulation and perfusion of the vital organs in the body, particularly in cardiac arrest patients who are not responsive to cCPR and fail to achieve the return of spontaneous circulation (ROSC) [35]. Previous studies including RCTs, and cohort studies have reported good neurological recovery, overall survival at discharge, and sustained ROSC in patients who underwent eCPR compared to cCPR [36–42]. One of the most critical determinants of neurological outcomes is low-flow duration. The minimization of low-flow duration can be achieved in hospital environments that are optimized for eCPR [43]. While reducing the low duration has better patient outcomes, performing eCPR too early may cause the patients to undergo an unnecessary and highly invasive intervention [43, 44]. 

Consistent with the results of our study, a 2022 systematic review and meta-analysis by Alfalasi et al. comparing cCPR with eCPR in OHCA patients reported significantly higher odds of favorable neurological outcomes at 3 and 6-months with eCPR; however, there were no significant differences between groups for survival outcomes and for favorable neurological outcomes at discharge and at 1 month [45]. Other recent meta-analyses likewise report eCPR to be associated with improved neurological outcomes at at least one follow-up interval, supporting a pattern of later benefit even when early endpoints are neutral [46–48].

Our findings suggest that while immediate neurological recovery may not show a statistically significant difference, the capacity of eCPR to sustain systemic perfusion and support vital organs over a longer period may enable better functional recovery later, an outcome that can be of greater importance to patients and clinicians. Neurological recovery after resuscitation is a complex, time-dependent process that may traverse phases of hyperemia, hypoperfusion, and eventual reperfusion/restoration of circulation; the full extent of neurological recovery and the benefits of eCPR may therefore require more time to manifest, which helps explain the non-significant findings at discharge and at 1-month in contrast with the significant improvements at 3 and 6-months.

Despite evidence pointing toward neurological (and in some studies survival) benefits of eCPR, clinicians should also account for the risk of acute brain injury in cardiac arrest patients undergoing eCPR. Mechanisms include reperfusion injury, reactive oxygen species generation, and microvascular dysregulation, all of which can mediate secondary brain injury after ROSC. Moreover, patients on peripheral cannulation for eCPR are at risk for Harlequin (north–south) syndrome [49]. Selective hypothermic cerebral perfusion has been proposed as a potential adjunct to mitigate brain injury by down-regulating neuroinflammation [50]. However, the literature remains limited regarding how pre-eCPR low-flow duration, circuit flow rates, and vasopressor use modify cerebral autoregulation and perfusion during and after eCPR, highlighting important knowledge gaps that warrant targeted investigation [51].

For survival to discharge, prior studies have reported mixed findings. Pagura et al., consistent with our results, observed significantly higher survival to discharge in the eCPR cohort [46]. In contrast, other studies did not find this association to reach statistical significance [47,48

### Limitations

Our study has some limitations. Firstly, many of our results showed significant heterogeneity. Potential sources of heterogeneity include differences in patient populations (e.g., age, comorbidities), eCPR initiation criteria and timing, operator expertise, and post-resuscitation care (e.g., targeted temperature management, hemodynamic optimization). Variations in study design, follow-up duration, and adjustment for confounders may also have contributed This was addressed through sensitivity analyses indicated significant changes in heterogeneity after the removal of outlier studies for favorable neurological outcomes at discharge and neurological outcomes at 1 month.

Moreover, the limited number of included studies prevented us from performing meta-regression analyses (> 15 studies required).Publication bias assessment through funnel plots could not be performed for all outcomes (> 10 studies required).

Lastly, majority of included studies being retrospective in nature may limit the generalizability of our findings.

## Conclusion

Our meta-analysis demonstrated that, among patients with OHCA, eCPR was associated with a statistically significant increase in survival to discharge and with higher odds of favorable neurological outcomes at discharge, 3 months, and 6 months. Taken together, these findings suggest that the provision of eCPR in the emergency management of OHCA may confer meaningful neurological and survival benefits and, consequently, may translate into improved long-term physiological status among survivors.

To ensure accurate estimation and interpretation of effects, future studies should adopt strict eligibility criteria, robust randomization, blinding where feasible, standardized treatment protocols, and careful monitoring of intervention fidelity to reduce heterogeneity. While the current evidence base still lacks sufficiently large, high-quality randomized data to permit definitive conclusions, this meta-analysis synthesizes the best available evidence to inform clinical decision-making and to guide the design of future trials. Well-powered, methodologically rigorous randomized trials are needed to validate these results.

## Supplementary Information

Supplementary Material 1

## Data Availability

The data presented in this meta-analysis are provided in this article/Supplementary material. Further inquiries can be directed to the corresponding author/s.
