# Supplementary material for: Extra-corporeal-cardiopulmonary-resuscitation vs. conventional-cardiopulmonary-resuscitation: an in-depth look into short- and long-term neurological outcomes
Source: J Cardiothorac Surg. 2025 Dec 24;20:465. doi: 10.1186/s13019-025-03708-z (PMC12729085; doi:10.1186/s13019-025-03708-z)

**Supplementary Table 1. PRISMA Checklist**

| **Section and Topic** | **Item #** | **Checklist item** | **Location where item is reported** |
| --- | --- | --- | --- |
| **TITLE** | | |  |
| Title | 1 | Identify the report as a systematic review. | Title Page |
| **ABSTRACT** | | |  |
| Abstract | 2 | See the PRISMA 2020 for Abstracts checklist. | Line 1-17 |
| **INTRODUCTION** | | |  |
| Rationale | 3 | Describe the rationale for the review in the context of existing knowledge. | Line 35-36 |
| Objectives | 4 | Provide an explicit statement of the objective(s) or question(s) the review addresses. | Line 36-37 |
| **METHODS** | | |  |
| Eligibility criteria | 5 | Specify the inclusion and exclusion criteria for the review and how studies were grouped for the syntheses. | Line 54-58 |
| Information sources | 6 | Specify all databases, registers, websites, organisations, reference lists and other sources searched or consulted to identify studies. Specify the date when each source was last searched or consulted. | Line 47-51 |
| Search strategy | 7 | Present the full search strategies for all databases, registers and websites, including any filters and limits used. | Supplementary Table 2 |
| Selection process | 8 | Specify the methods used to decide whether a study met the inclusion criteria of the review, including how many reviewers screened each record and each report retrieved, whether they worked independently, and if applicable, details of automation tools used in the process. | Line 52-58 |
| Data collection process | 9 | Specify the methods used to collect data from reports, including how many reviewers collected data from each report, whether they worked independently, any processes for obtaining or confirming data from study investigators, and if applicable, details of automation tools used in the process. | Line 67-69 |
| Data items | 10a | List and define all outcomes for which data were sought. Specify whether all results that were compatible with each outcome domain in each study were sought (e.g. for all measures, time points, analyses), and if not, the methods used to decide which results to collect. | Line 60-65 |
|  | 10b | List and define all other variables for which data were sought (e.g. participant and intervention characteristics, funding sources). Describe any assumptions made about any missing or unclear information. | Line 68-69 |
| Study risk of bias assessment | 11 | Specify the methods used to assess risk of bias in the included studies, including details of the tool(s) used, how many reviewers assessed each study and whether they worked independently, and if applicable, details of automation tools used in the process. | Line 70-72 |
| Effect measures | 12 | Specify for each outcome the effect measure(s) (e.g. risk ratio, mean difference) used in the synthesis or presentation of results. | Line 70-77 |
| Synthesis methods | 13a | Describe the processes used to decide which studies were eligible for each synthesis (e.g. tabulating the study intervention characteristics and comparing against the planned groups for each synthesis (item #5)). | Table.1 |
|  | 13b | Describe any methods required to prepare the data for presentation or synthesis, such as handling of missing summary statistics, or data conversions. | N/A |
|  | 13c | Describe any methods used to tabulate or visually display results of individual studies and syntheses. | Figure 2-3 |
|  | 13d | Describe any methods used to synthesize results and provide a rationale for the choice(s). If meta-analysis was performed, describe the model(s), method(s) to identify the presence and extent of statistical heterogeneity, and software package(s) used. | Line 74-77 |
|  | 13e | Describe any methods used to explore possible causes of heterogeneity among study results (e.g. subgroup analysis, meta-regression). | N/A |
|  | 13f | Describe any sensitivity analyses conducted to assess robustness of the synthesized results. | Line 78-79  Supplementary Table 5,6  Supplementary figure 3 |
| Reporting bias assessment | 14 | Describe any methods used to assess risk of bias due to missing results in a synthesis (arising from reporting biases). | Not applicable |
| Certainty assessment | 15 | Describe any methods used to assess certainty (or confidence) in the body of evidence for an outcome. | Line 89-90 |
| **RESULTS** | | |  |
| Study selection | 16a | Describe the results of the search and selection process, from the number of records identified in the search to the number of studies included in the review, ideally using a flow diagram. | Line 83-86  Figure.1 |
|  | 16b | Cite studies that might appear to meet the inclusion criteria, but which were excluded, and explain why they were excluded. | Figure.1 |
| Study characteristics | 17 | Cite each included study and present its characteristics. | Line 83  Table.1 |
| Risk of bias in studies | 18 | Present assessments of risk of bias for each included study. | Line 88  Supplementary Figure 1 and Supplementary Table 3 |
| Results of individual studies | 19 | For all outcomes, present, for each study: (a) summary statistics for each group (where appropriate) and (b) an effect estimate and its precision (e.g. confidence/credible interval), ideally using structured tables or plots. | Figure.2-3 |
| Results of syntheses | 20a | For each synthesis, briefly summarise the characteristics and risk of bias among contributing studies. | Table 1  Supplementary Figure 1 and Supplementary Table 3 |
|  | 20b | Present results of all statistical syntheses conducted. If meta-analysis was done, present for each the summary estimate and its precision (e.g. confidence/credible interval) and measures of statistical heterogeneity. If comparing groups, describe the direction of the effect. | Line 94-109  Figures 2-3 |
|  | 20c | Present results of all investigations of possible causes of heterogeneity among study results. | Line 146-169  Supplementary Table 4  Supplementary Figure 2-12 |
|  | 20d | Present results of all sensitivity analyses conducted to assess the robustness of the synthesized results. | Line 110-112  Supplementary Table 5,6  Supplementary figure 3 |
| Reporting biases | 21 | Present assessments of risk of bias due to missing results (arising from reporting biases) for each synthesis assessed. | Line 91-92 (supplementary figure 2) |
| Certainty of evidence | 22 | Present assessments of certainty (or confidence) in the body of evidence for each outcome assessed. | Line 89=90  Supplementary Figure 4 |
| **DISCUSSION** | | |  |
| Discussion | 23a | Provide a general interpretation of the results in the context of other evidence. | Line 126-129 |
|  | 23b | Discuss any limitations of the evidence included in the review. | Line 148-152,155 |
|  | 23c | Discuss any limitations of the review processes used. | Line 153-154 |
|  | 23d | Discuss implications of the results for practice, policy, and future research. | Line 157-164 |
| **OTHER INFORMATION** | | |  |
| Registration and protocol | 24a | Provide registration information for the review, including register name and registration number, or state that the review was not registered. | 43-44 |
|  | 24b | Indicate where the review protocol can be accessed, or state that a protocol was not prepared. | 43 |
|  | 24c | Describe and explain any amendments to information provided at registration or in the protocol. | - |
| Support | 25 | Describe sources of financial or non-financial support for the review, and the role of the funders or sponsors in the review. | N/A |
| Competing interests | 26 | Declare any competing interests of review authors. | Title page |
| Availability of data, code and other materials | 27 | Report which of the following are publicly available and where they can be found: template data collection forms; data extracted from included studies; data used for all analyses; analytic code; any other materials used in the review. | N/A |

**Supplementary Table 2. Search Strategy**

| **Database** | **Search Strategy** |
| --- | --- |
| PubMed/MEDLINE  (935 results) | "out-of-hospital"[All Fields] AND ("cardiopulmonary resuscitation"[All Fields] OR "cardiopulmonary arrest"[All Fields] OR "cardiac arrest"[All Fields]) AND ("ECPR"[All Fields] OR "extracorporeal cardiopulmonary resuscitation"[All Fields] OR "extracorporeal support"[All Fields] OR "extracorporeal membrane oxygenation"[All Fields]) |
| Cochrane Library  (92 results) | (out-of-hospital) AND (cardiopulmonary resuscitation OR cardiopulmonary arrest OR cardiac arrest) AND (ECPR OR extracorporeal cardiopulmonary resuscitation OR extracorporeal support OR extracorporeal membrane oxygenation) |
| SCOPUS  (1,140 results) | (“out-of-hospital”) AND (“cardiopulmonary resuscitation” OR “cardiopulmonary arrest” OR “cardiac arrest”) AND (“ECPR” OR “extracorporeal cardiopulmonary resuscitation” OR “extracorporeal support” OR “extracorporeal membrane oxygenation”) |

**Supplementary Table 3. Newcastle Ottawa Scale Quality Assessment results.**

|  | Bougouin et al. 2020 | Choi et al. 2016 | Choi et al. 2016 | Kim et al. 2014 | Kitada et al. 2020 | Maekawa et al. 2013 | Nakashima et al. 2019 | Patricio et al. 2019 | Sakamoto et al. 2014 | Schober et al. 2017 | Yannopoulos et al. 2017 | Yoshida et al. 2020 | Jeong et al. 2022 | Verdonschot et al. 2024 | Sun et al. 2023 | Shih 2024 | Lee 2025 |
| --- | --- | --- | --- | --- | --- | --- | --- | --- | --- | --- | --- | --- | --- | --- | --- | --- | --- |
| **Selection (4)** |  |  |  |  |  |  |  |  |  |  |  |  |  |  |  |  |  |
| Representativeness of the exposed cohort | * | * | * | * | * | * | * | * | * | * | * | * | * | * | * | * | * |
| Selection of the non-exposed cohort | * | * | * | * | * | * | * | * | * | * | * | * | * | * | * | * | * |
| Ascertainment of exposure | * | * | * | * | * | * | * | * | * | * |  |  |  | * | * | * | * |
| Demonstration that outcome of interest was not present at start of study | * | * | * | * | * | * | * | * |  |  | * | * | * | * | * |  |  |
| **Comparability (2)** |  |  |  |  |  |  |  |  |  |  |  |  |  |  |  |  |  |
| Comparability of cohorts on the basis of the design or analysis | * | ** | ** | * | ** | ** | ** | ** | * | ** | * | * | ** | ** | * | ** | ** |
| **Outcome (3)** |  |  |  |  |  |  |  |  |  |  |  |  |  |  |  |  |  |
| Assessment of outcome | * |  | * | * | * | * | * |  | * | * | * | * | * |  | * | * | * |
| Was follow-up long enough for outcomes to occur | * |  |  | * | * | * | * | * | * | * | * | * | * |  | * | * | * |
| Adequacy of follow up of cohorts | * | * | * | * | * | * | * | * | * | * | * | * | * | * | * | * | * |
| **Total (9)** | 8 | 7 | 8 | 8 | 9 | 9 | 9 | 8 | 7 | 8 | 7 | **7** | **8** | **7** | **8** | **8** | **8** |
| **Interpretation** | Low risk | Low risk | Low risk | Low risk | Low risk | Low risk | Low risk | Low risk | Low risk | Low risk | Low risk | Low risk | Low risk | Low risk | Low risk | Low risk | Low risk |

**Supplementary Table 4. GRADE assessment**

| **GRADE assessment:** Neurological outcomes of eCPR vs cCPR in OHCA | | | | | |
| --- | --- | --- | --- | --- | --- |
| **People: Patients with out-of-hospital cardiac arrest**  **Settings:** EMS and hospital  **Intervention:** eCPR  **Comparison:** cCPR | | | | | |
| **Outcomes** | **Absolute Effect** | | **Relative effect**  **(95% CI)** | **Number of studies** | **Certainty of the evidence (GRADE)^†^** |
|  | cCPR | eCPR |  |  |  |
| Favorable neurological outcome at discharge | **69 per 1000** | **138 per 1000** | OR 2.61 (95% CI 1.28 to 5.32) | 1 RCTs  6 observational studies | ⊕⊕⊕⊕ High |
|  | **+69 per 1000** (95% CI +19 to +298) | |  |  |  |
| **Favorable neurological outcome at 1-month** | **130 per 1000** | **195 per 1000** | OR 2.15 (95% CI 0.87 to 5.34) | 2 RCTs  5 observational studies | ⊕⊕⊕⊕ High |
|  | **+65 per 1000** (95% CI: –17 to +350 per 1000) | |  |  |  |
| **Favorable neurological outcome at 3-months** | **42 per 1000** | **80 per 1000** | OR 3.29 (95% CI 1.63 to 6.63) | 2 RCTs  5 observational studies | ⊕⊕⊕⊕ High |
|  | **+38 per 1000** (95% CI: +20 to +81) | |  |  |  |
| **Favorable neurological outcome at 6-months** | **82 per 1000** | **156 per 1000** | OR 1.97 (95% CI 1.24 to 3.12) | 2 RCTs  3 observational studies | ⊕⊕⊕⊕ High |
|  | **+74 per 1000** (95% CI +20 to +174) | |  |  |  |
| **Survival-to-hospital discharge** | 86 per 1000 | 146 per 1000 | OR 1.84 (95% CI 1.17 to 2.92) | 1 RCT  9 observational studies | ⊕⊕⊕⊖ Moderate |
|  | **+60 per 1000** (95% CI +15 to +165) | |  |  |  |
| 95% CI: 95% Confidence interval; OR: Odds ratio  **^†^** GRADE Working Group grades of evidence  **High** = This research provides a very good indication of the likely effect. The likelihood that the effect will be substantially different^‡^ is low.  **Moderate** = This research provides a good indication of the likely effect. The likelihood that the effect will be substantially different^‡^ is moderate.  **Low** = This research provides some indication of the likely effect. However, the likelihood that it will be substantially different^‡^ is high.  **Very low** = This research does not provide a reliable indication of the likely effect. The likelihood that the effect will be substantially different^‡^ is very high.  ^‡^ Substantially different = a large enough difference that it might affect a decision | | | | | |

**Supplementary Table 5.**

**Leave-One-Out Sensitivity Analysis (Neurological Outcomes at discharge)**

| **Study omitted** | **Pooled OR (95% CI)** | **I² (%)** | **Notes** |
| --- | --- | --- | --- |
| None (all studies included) | 2.61 (1.28–5.32) | 82 | Reference (all studies) |
| Kim et al. 2014 | 2.36 (1.14–4.89) | 83 | Minimal effect on heterogeneity |
| Choi et al. 2016 | 3.05 (1.23–7.57) | 85 | Stronger effect but worse heterogeneity |
| Yannopoulos et al. 2017 | 2.38 (1.09–5.18) | 80 | Modest reduction in heterogeneity |
| Bougoin et al. 2020 | 3.21 (1.91–5.41) | 44 | Major source of heterogeneity |
| Yannopoulos et al. 2020 | 2.47 (1.19–5.11) | 84 | Minimal effect |
| Sun, Peng et al. 2023 | 2.72 (1.22–6.08) | 85 | No improvement |
| Lee et al. 2025 | 2.22 (1.08–4.60) | 77 | Some heterogeneity contribution |
|  |  |  |  |
| **Supplementary Table 6.**  **Leave-One-Out Sensitivity Analysis (1-Month Neurological Outcomes)** | | | |
| **Study omitted** | **Pooled OR (95% CI)** | **I² (%)** | **Notes** |
| **None (all studies included)** | 2.15 (0.87–5.34) | 90 | Reference (all studies) |
| Choi et al. 2016 (2) | 1.81 (0.71–4.61) | 91 | Minimal effect on heterogeneity |
| Sakamoto et al. 2014 | 1.70 (0.68–4.25) | 90 | Slight reduction in effect size |
| Kitada et al. 2020 | 2.97 (1.22–7.22) | 84 | Stronger effect, modest reduction in heterogeneity |
| Yoshida et al. 2020 | 1.62 (0.68–3.87) | 89 | Lower effect, modest heterogeneity reduction |
| Jeong et al. 2022 | 2.75 (0.84–9.02) | — | Stronger effect, heterogeneity not reported |
| Belohlavek et al. 2022 | 2.24 (0.76–6.63) | 91 | Slightly stronger effect, minimal heterogeneity change |
| Suveiren et al. 2023 | 2.39 (0.84–6.83) | 92 | Stronger effect but worse heterogeneity |
|  |  |  |  |

**Supplementary Figure 1. Risk of Bias Quality Assessment of Non-Randomized Studies**


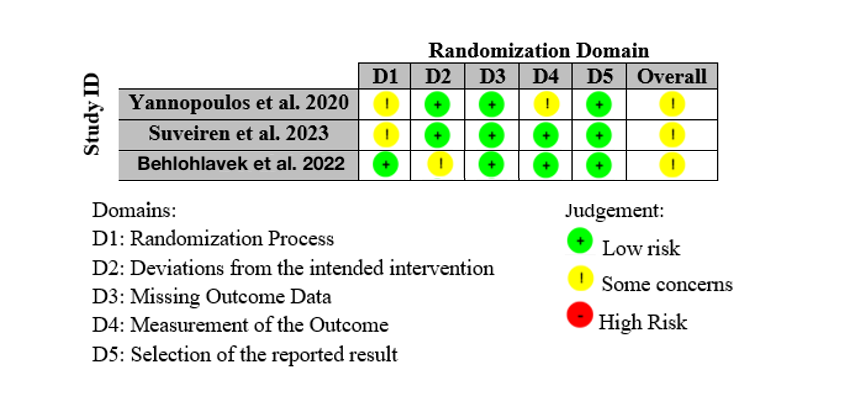


**Supplementary Figure 2. Publication Bias – Survival-to-discharge**

**
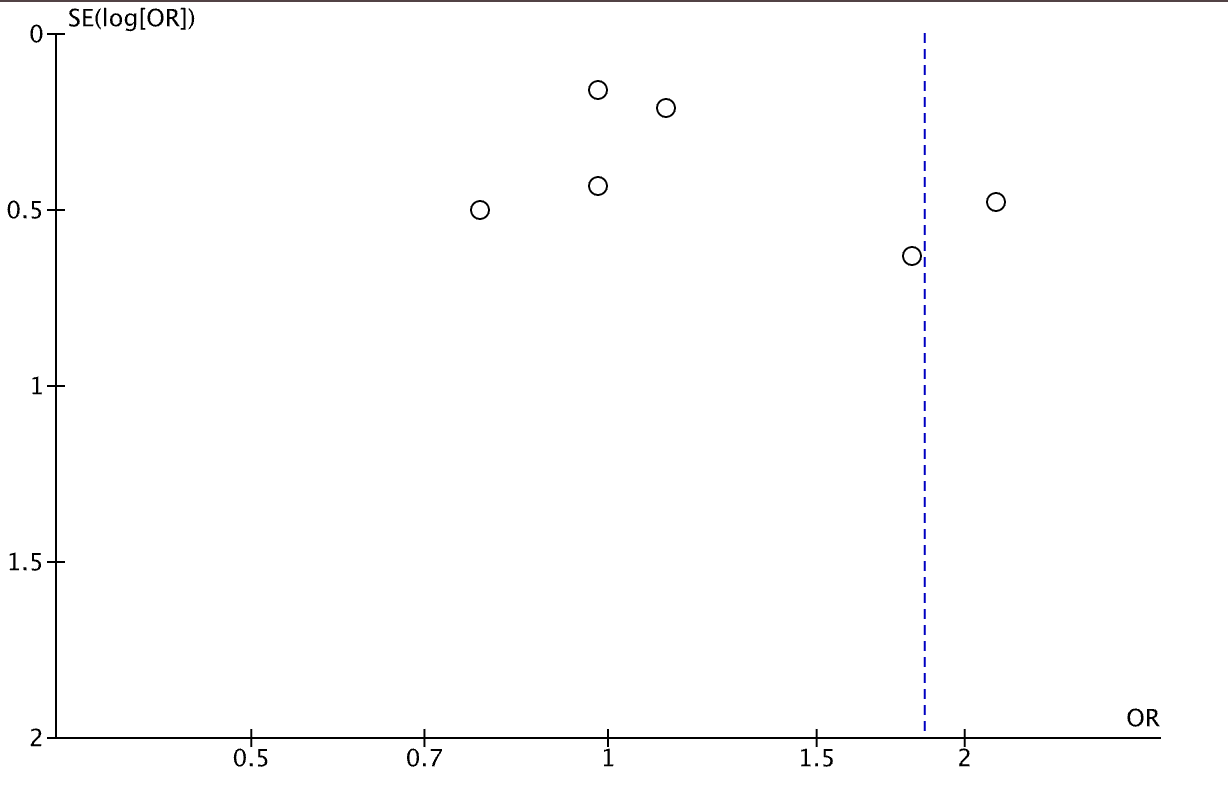
**

**Supplementary Figure 3. Sensitivity Analysis of Neurological Outcomes at 1, 3, 6-months.**


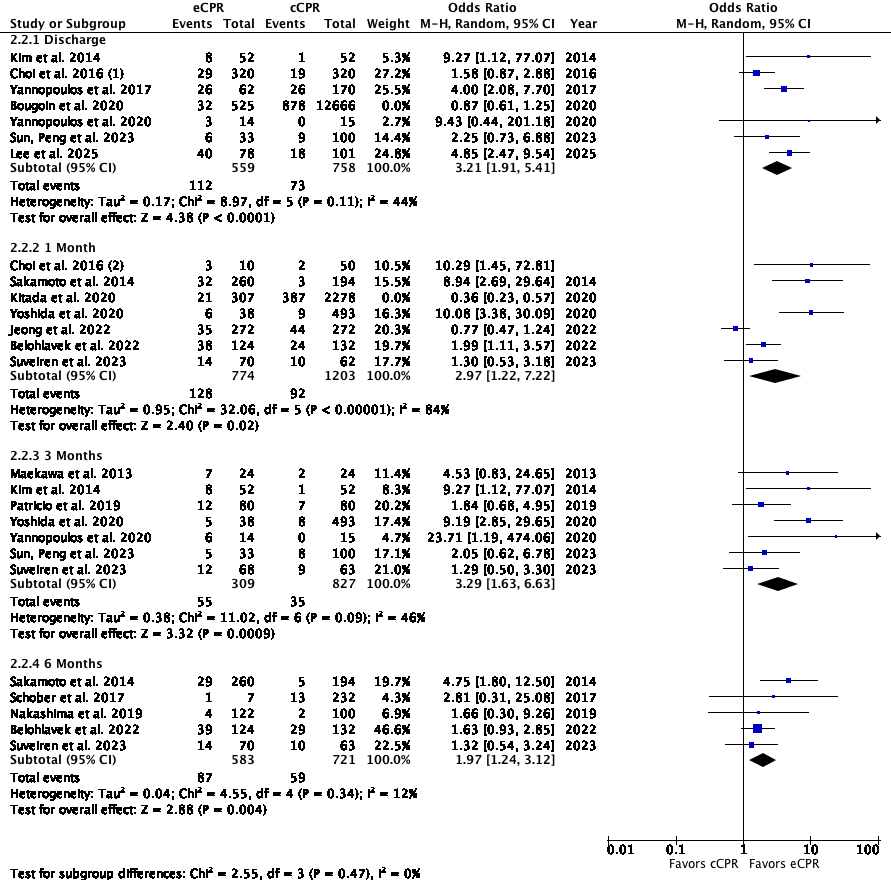

Supplement: Supplementary file 1 — Supplementary Material 1 [file 13019_2025_3708_MOESM1_ESM.docx]
